# Specificity and longevity of a bacterial interspecies mutual cooperation benefiting organic micropollutant biodegradation

**DOI:** 10.1128/aem.00093-26

**Published:** 2026-07-01

**Authors:** Siyao Du, Benjamin Horemans, Dirk Springael

**Affiliations:** 1 Division of Soil and Water Management, KU Leuven, Heverlee, Belgium; Danmarks Tekniske Universitet, Kgs. Lyngby, Denmark

**Keywords:** drinking water treatment, bioaugmentation, micropollutant biodegradation, accidental cooperative interaction

## Abstract

*Aminobacter niigataensis* MSH1 is a candidate for bioaugmentation of sand filters in drinking water treatment plants (DWTP), as it mineralizes the ubiquitous groundwater micropollutant 2,6-dichlorobenzamide (BAM). The DWTP sand filter isolate *Piscinibacter* sp. K169 improves BAM mineralization by MSH1 in an apparent accidental mutual cooperation, and co-inoculation of the organism was proposed to assist bioaugmentation with MSH1. In this study, we questioned whether this accidental mutual positive interaction extends to four other pesticide catabolic bacterial strains of the same or a different genus of MSH1, and examined the longevity of the cooperation. Negative interactions were never observed in either direction. As observed for BAM mineralization by MSH1, K169 stimulated BAM mineralization by *A. niigataensis* LG1 and 2,4-D mineralization by *Cupriavidus pinatubonensis* JMP134 without affecting the cell density of the catabolic strains. Linuron mineralization by *Variovorax* sp. SRS16 and carbofuran mineralization by *Novosphingobium* sp. KN65.2 were not affected. In the other direction, growth of K169 was stimulated by all pesticide catabolic strains except JMP134, indicating a common underlying mechanism. After 2 weeks, the beneficial effects of K169 on MSH1, LG1, and JMP134 functionality diminished or even reversed, likely because of organic carbon depletion. In contrast, cell densities of K169 in all dual-species systems remained higher than in the K169 monoculture system. This study extends our knowledge on accidental interactions and the beneficial effect of a sand filter isolate toward other pesticide degraders, opening doors for *Piscinibacter* sp. K169-assisted bioaugmentation of other/multiple pesticide degraders in DWTPs.

**IMPORTANCE:**

Sand‑filter bioaugmentation with the BAM‑catabolic *Aminobacter niigataensis* MSH1 represents an advanced strategy for removing BAM from groundwater in drinking water treatment; however, prior studies indicate that efficacy lasts only for 1–2 weeks. *Piscinibacter* sp. K169, an isolate from drinking‑water sand filters, supports mineralization of BAM by MSH1 through accidental mutual cooperation, and co‑inoculation with K169 was suggested as an innovation to improve MSH1 bioaugmentation. We show that K169 promotes mineralization of OMPs by other bacteria and, hence, that the K169-degrader cooperation can be extended to support removal of other or even multiple OMPs. Benefits declined over time, likely due to nutrient depletion, making nutrient management a requirement for maintaining the cooperation. To the best of our knowledge, this is the first study to examine specificity in accidental microbial cooperation, especially in a bioaugmentation context of water treatment. It is relevant both to a fundamental understanding of accidental microbial interactions and to applications in water treatment.

## INTRODUCTION

Organic micropollutants (OMPs) like pesticides and their transformation products are pollutants that occur at trace concentrations (ng/L–µg/L range) in environmental freshwaters, including source waters for drinking water production (1). In the European Union, the threshold concentration of pesticide residues in drinking water is 0.1 μg/L for an individual compound and 0.5 μg/L for the sum of the compounds (2). Often, the concentration of OMPs in drinking water resources exceeds the regulatory thresholds, and the water needs to be treated before it is released into the municipal distribution network, posing a major challenge for drinking water treatment plants (DWTPs). Bioaugmentation, that is, the introduction of specific OMP/pesticide-degrading bacteria to a treatment module, such as rapid sand filters in DWTPs, is proposed as a cost-efficient and sustainable alternative treatment approach for expensive and less sustainable physico-chemical treatment options (3–5).

2,6-dichlorobenzamide (BAM) is a highly recalcitrant transformation product of the widely used herbicide dichlobenil (2,6-dichlorobenzonitrile) and a ubiquitous OMP, especially in groundwater (6–9). *Aminobacter niigataensis* MSH1, isolated from soil, mineralizes BAM at micro-concentrations as low as 1.5 μg/L (10–12). The organism has been successfully used for bioaugmentation of sand filters to treat BAM-contaminated water in laboratory and pilot DWTP sand filters, showing a decrease in BAM concentration in groundwaters from 0.4 μg/L to below the 0.1 μg/L threshold (13–15). However, BAM biodegradation deteriorated between 5 and 11 days after MSH1 inoculation, likely due to the loss of cell density and activity linked with the depletion of nutrient and energy resources inherent to the oligotrophic DWTP environment (13, 14, 16).

Increasing evidence suggests that microbial interactions between non-degraders and pesticide-degrading bacteria can strongly influence the efficiency and stability of pesticide degradation processes within a microbial community (17–20). *Piscinibacter* sp. K169, isolated from a DWTP rapid sand filter unit (21), was shown to interact with MSH1 cells in sand microcosms by improving its BAM mineralizing functionality, while MSH1 stimulated the growth of K169. Apparently, the two organisms engage in mutual cooperation, resulting in improved BAM biodegradation (22). Based on these observations, a new bioaugmentation concept was proposed in which bioaugmentation with a pollutant degrader, like *A. niigataensis* MSH1, is assisted by co-inoculation of a strain that is adapted to the target environment and supports the degrader strain, as is the case for *Piscinibacter* sp. K169 (22). The mutual positive interaction between K169 and MSH1 is between two independently isolated organisms derived from different environments and can be considered accidental (23). While such assemblies have been shown before to engage in mutual facilitations (24–26), it is not clear whether and to what extent one of the partners can engage in similar interactions with other bacteria and hence questions the specificity of the accidental interaction. This question is of particular relevance for the above-mentioned biotechnological concept of using “assistant” bacteria to support/improve bioaugmentation with a degrader organism, since it would be of interest that the assistant organism also stimulates other bacteria degrading other OMPs beyond BAM for bioaugmentation purposes. Therefore, the current study aims to acquire an understanding of the specificity of the mutual positive interactions that were previously observed between *Piscinibacter* sp. K169 and *A. niigataensis* MSH1, that is, whether *Piscinibacter* sp. K169 can also engage in stimulating pesticide degradation of other organisms beyond MSH1 and, if so, whether a mutual positive relationship is involved in which the pesticide degrader also stimulates growth of K169. Moreover, we assessed the longevity of the interactions, including mutual cooperation. Recently, the main carbon source that mediates the mutual positive interaction between MSH1 and K169 was shown to be the organic carbon present on the sand (27). We hypothesize that this carbon or any other key nutrient is exhaustive and, hence, that the mutual cooperative interaction might be time-dependent and dependent on the involved organisms. The knowledge about interactions in the longer term is of special interest for bioaugmentation in DWTPs, of which one of the challenges is to maintain biodegradation activity toward the target pesticide over extended time scales (28).

To test these hypotheses, dual-species bacterial assemblies consisting of one of five different pesticide catabolic strains and K169 were incubated in coarse sand microcosms, mimicking the oligotrophic DWTP sand filter environment, allowing interactions to come into play, as done previously for studying the interaction between MSH1 and K169 (22). The impact of co-existence of the two strains on pesticide mineralization by the pesticide-catabolic strains and viability/growth of both strains was determined after a 7-day incubation phase by adding ^14^C-labeled pesticide and monitoring ^14^CO_2_ production kinetics and by determining cell densities, respectively, as compared to monoculture systems to evaluate the nature of the interaction between K169 and the respective pesticide catabolic strain. This 7-day time period between inoculation and determining pesticide mineralization activity and cell densities was chosen because previous research demonstrated that between 5 and 11 days after inoculation, MSH1 functionality in sand filter systems started to deteriorate (13–15). In addition, in the current study, we also examined the nature of the interaction after a 14-day incubation period to examine its longevity. The tested pesticide catabolic strains included beyond MSH1, *Cupriavidus pinatubonensis* JMP134 which uses the chlorophenoxy herbicide 2,4-dichlorophenoxyacetic acid (2,4-D) as the sole C source (29), *Variovorax* sp. SRS16 metabolizing the phenyl urea herbicide linuron (3-(3,4-dichlorophenyl)−1-methoxy-1-methylurea) (30), *Novosphingobium* sp. KN65.2 metabolizing the carbamate insecticide carbofuran (2,3-dihydro-2,2-dimethylbenzofuran-7-yl methylcarbamate) (31), and *A. niigataensis* LG1 metabolizing BAM. Strains JMP134, SRS16, and KN65.2 are often used as model organisms to study different microbial and ecological aspects of pesticide degradation; they degrade three different types of OMPs/pesticides found in groundwater and belong to three different genera. Strain LG1 is taxonomically very close to MSH1, degrades the same compound, and therefore is hypothesized to have the highest likelihood to show interactions similar to those observed between K169 and MSH1.

## MATERIALS AND METHODS

### Bacterial strains and growth conditions

A green fluorescent protein (GFP)-labeled and kanamycin (Km)-resistant variant of *A. niigataensis* MSH1, that is, MSH1-GFP, was used (16). The BAM catabolic *A. niigataensis* LG1 was isolated from soil sampled from a roadside in Wespelaar, Belgium, that was regularly treated with the herbicide dichlobenil. The organism was recovered by a classic enrichment procedure in MMO medium containing 200 mg/L BAM; it mineralizes BAM and is naturally resistant to Km at a concentration of 50 mg/L. The other pesticide catabolic bacterial strains *Cupriavidus necator* JMP134, *Novosphingobium* sp. KN65.2, and *Variovorax* sp. SRS16 were described before (29–31). In the case of *Piscinibacter* sp. K169, the rifampicin (Rif)-resistant variant, that is, K169-RIF, allowing specific counting on rifampicin-containing media, was used as described before (22). The strains were routinely grown from frozen stocks preserved at −80°C by plating on suitable media and incubation at 25°C. Subsequently, a smear of colonies was inoculated in 50 mL liquid medium, and the cultures were incubated at 25°C on an orbital shaker at 180 rpm till an OD600 of 1.0 was reached. Used growth media and time period of incubation differed for each strain and are listed in Table S1.

### Microcosm setup

Sand microcosms were set up as described by Vandermaesen et al. (22) as dual-species assemblies (*R_T_* = 2) consisting of K169 and one of each of the pesticide catabolic strains, and as mono-species systems (*R_T_* = 1) of K169 and of each of the pesticide catabolic bacteria. Specifically, microcosms were set up in deep 96-well plates (Thermo Scientific Nunc, Waltham, MA, USA) containing 150 mg washed and sterilized sand in each well and 100 μL cell suspension at a cell density of 10^7^ cells/mL for each strain in MMO medium (32) amended with 150 μg/L Na-acetate. The different organisms were precultured as described by Vandermaesen et al. (22). The precultures were washed three times by centrifugation (4,000 × *g*, 15 min, 15℃) before resuspension in the MMO medium containing acetate. Cell densities were determined by flow cytometry of live/dead-stained cells using an Accuri C6 Cytometer (BD Biosciences, East Rutherford, NJ, USA) and adjusted based on the live cell density as described previously (22). To improve the staining of *Variovorax* sp. SRS16 cells, Na-EDTA was added at a final concentration of 0.05 mM to the suspension. Abiotic (uninoculated) controls (*R_T_* = 0), that is, microcosms without bacteria, were included to score background mineralization of the target pesticides. Four replicates were always included for each strain combination, single-strain system, and the abiotic control. The whole setup was prepared in quadruple to allow analysis at two different time points either for assessing pesticide mineralization or determining cell densities. After sealing, the microcosm systems were incubated at 20℃ for a period of either 7 days (t_7_) and 14 days (t_14_). At t_7_ and t_14_, using two of the four setups and depending on the used catabolic strain, 5,000 counts per minute (cpm) of [benzene ring-U−^14^C]-labeled BAM, linuron, carbofuran, or 2,4-D (> 95% purity; Institute of Isotopes Co., Ltd., Budapest, Hungary) dissolved in 5 μL MMO was spiked in each microcosm well corresponding to a concentration of 150, 71, 162, and 123 μg/L, respectively. Pesticide mineralization was subsequently monitored by determining ^14^CO_2_ production from the respective ^14^C-labeled pesticide at 19 time points during 130 h, that is, at 0.25, 0.75, 1.25, 1.75, 2.25, 2.75, 3.25, 3.75, 4.25, 4.75, 7.25, 8.25, 9.5, 10.75, 11.75, 14.75, 25.75, 50.5, 99.25, and 130 h after adding the ^14^C-labeled compound. ^14^CO_2_ production was determined by capturing ^14^CO_2_ in the wells by means of small cellulose filters impregnated with Ca(OH)_2_ and placed on an adhesive sealing tape in a pattern corresponding to the microtiter-plate wells as described previously (33). At each time point, the adhesive tape with the cellulose filters was replaced with a fresh one, and the captured ^14^CO_2_ was quantified by autoradiography combined with digital image analysis using CRMD4.0 General screens (Agfa HealthCare NV, Mortsel, Belgium) and a Typhoon 9400 scanner (Amersham Biosciences, Piscataway, NJ, USA) (33). Abiotic controls (*R_T_* = 0) showed no ^14^CO_2_ production. Cumulative mineralization curves were obtained by plotting the cumulative percentage ^14^CO_2_ (summing up the values determined at each time point) relative to the total amount of ^14^C added as a function of incubation time (22). Pesticide mineralization kinetic parameters *λ*, *µ,* and *A* were obtained by fitting the curve to the modified Gompertz model (34) using the following formula:


$$P\left(t\right)=A\;\;exp\left(-exp\left(\frac{e}{A}\left(\mu \lambda -\left(\mu -c\right)t\right)+1\right)\right)+\;ct$$

in which *P* (%) refers to the percentage mineralization at time *t* (h), *A* (%) is the total extent of mineralization after the exponential mineralization phase, *λ* (h) is the lag time, *µ* (% h^−1^) is the maximum mineralization rate constant, and *c* (% h^−1^) is the endogenous mineralization rate constant. Lsqnonlin command in Matlab R2012b (Mathworks, Natick, MA, USA) was used for parameter determination. As explained before for the MSH1/K169 interaction (22), the effect of K169 on pesticide mineralization and the nature of the interaction (positive, neutral, or negative) was scored based on *λ* and *µ*. Significantly (95% significance level) decreased *λ* and/or increased *µ* in dual-species assemblies compared to those of mono-species systems were scored as a positive interaction, while significantly increased *λ* and/or decreased *µ* were scored as a negative interaction. No effects on *λ* and *µ* were defined as neutral interactions. *A* was not directly considered to evaluate the effect on pesticide mineralization as explained before (22), while parameter *c* was not considered since its value was always close to zero. At t_7_ and t_14_, the cells in the microcosm wells were extracted from one of the two residual setups as described before (22), and the cell densities of each strain determined as CFU by plating tenfold dilution series on appropriate selective media R2A + Rif (50 mg/L) for counting K169; R2A + BAM (100 mg/L) + Km (50 mg/L) for counting MSH1 and LG1; MMO + glucose (2 g/L) for counting JMP134 and KN65.2; and MMO + tryptone (0.5 g/L) for counting SRS16 and incubation of the plates at 25°C (22). Cell densities at t_7_ and t_14_ were denoted as *D_t7,X_* and *D_t14,X_*, respectively, where *X* refers to the examined strain.

### Statistics

Significant differences between *λ*, *µ*, *A*, *D_t7,X_*, and *D_t14,X_* values (*n* = 4) between dual- and mono-species systems were determined using the pairwise Tukey test (35) with the IBM SPSS Statistics 22. The difference was evaluated as significant when the *P*-value was <0.05.

## RESULTS

### Pesticide mineralization and cell densities in dual-species versus mono-species systems at t_7_

**Fig 1:**
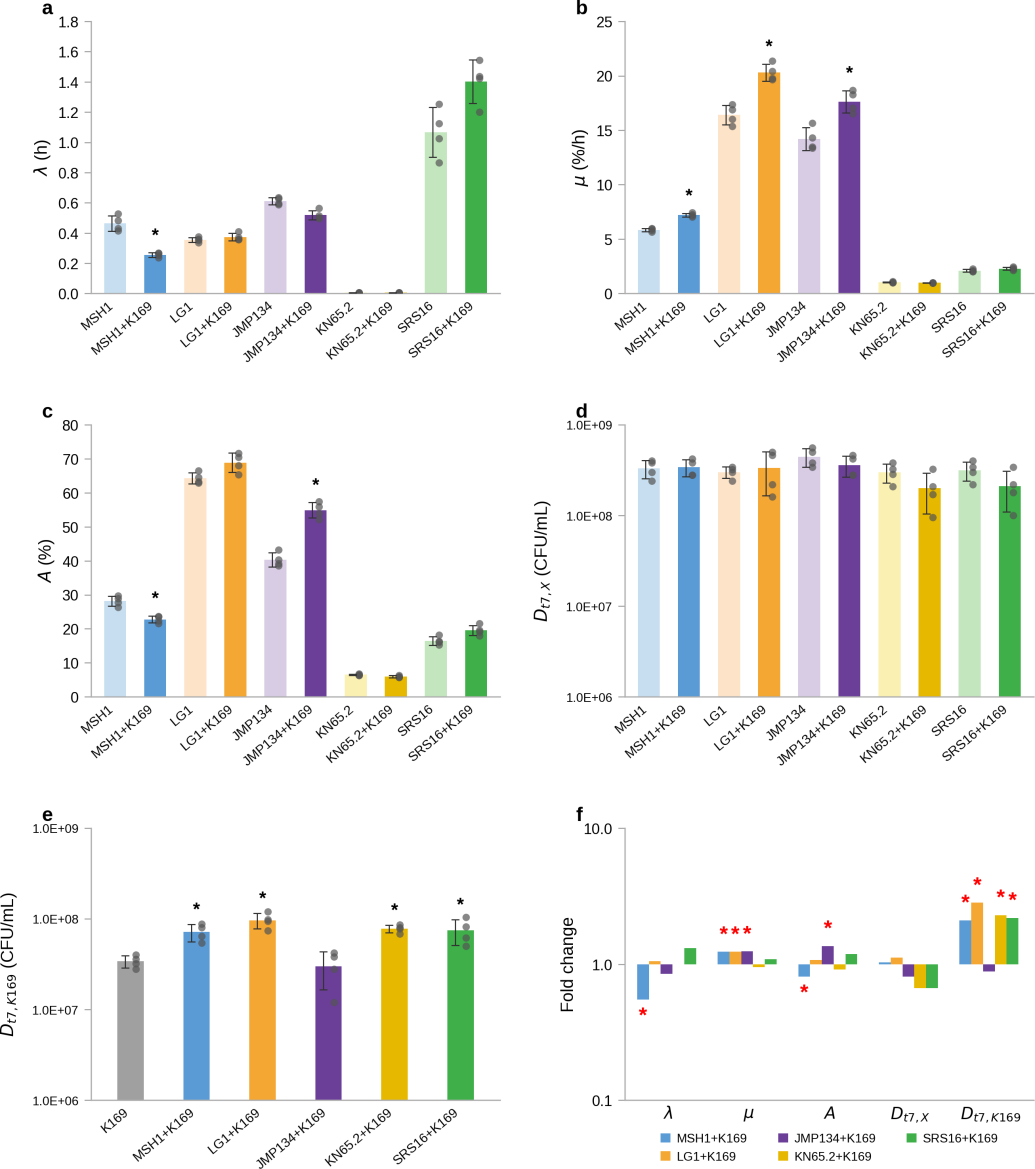
Values of pesticide mineralization parameters lag time (*λ*, panel **a**), maximum rate (*µ*, panel **b**), and extent (*A*, panel **c**) and cell densities of the tested pesticide catabolic bacteria (*D_t7,X_*, panel **d**) and K169 (*D_t7,K169_*, panel **e**) at t_7_ as observed in dual-species assemblies (*R_T_* = 2) of a pesticide catabolic strain and *Piscinibacter* sp. K169 and in respective mono-species systems (*R_T_* = 1). Panel **f** shows the fold change of the respective parameters in the dual-species systems (*R_T_* = 2) compared to the respective mono-species systems (*R_T_* = 1). The values shown are averages with standard deviation (shown in error bars) based on four replicates, with the dots showing the values of each replicate. The black asterisk in panels a to e indicates significant differences between *R_T_* = 2 and *R_T_* = 1 systems containing the same degrader strain and/or K169 (*P*-value < 0.05). The red asterisk in panel f indicates values for which the difference between *R_T_* = 2 and *R_T_* = 1 systems was significant (*P*-value < 0.05). The color of each bar corresponds to those used for indicating the mineralization curves in Fig. S1 from which the respective parameter values were deduced.

To evaluate whether K169 displays positive interactions with pesticide catabolic bacteria other than MSH1, the effect of K169 on pesticide mineralization and cell densities in each mono- and dual-species microcosm setup for MSH1, LG1, JMP134, KN65.2, and SRS16 was examined after 7 days of interaction. Mineralization kinetics are shown in Fig. S1. Values of *λ*, *µ*, *A*, and *D_t7,X_* are shown in Table S2 and graphically in Fig. 1a through e. Calculated fold changes in the respective parameters between dual-species systems and relevant mono-species systems are shown in Table S3 and Fig. 1f. As observed previously (22), BAM mineralization mediated by MSH1 was positively affected when K169 was present, as demonstrated by the shorter lag time (*λ*) and the higher maximum mineralization rate (*μ*) (Fig. 1a and b) in the dual-species assembly compared to the MSH1 mono-species system. K169 displayed a positive effect on BAM mineralization by LG1 and 2,4-D mineralization by JMP134, but only due to a higher *μ* value, while *λ* remained unchanged compared to the respective mono-species systems (Fig. 1a and b). Opposite to the lower *A* value of BAM mineralization by MSH1 in the dual-species system compared to the MSH1 monoculture, 2,4-D mineralization by JMP134 showed a higher *A* value in the presence of K169 (Fig. 1c). On the other hand, the presence of K169 was neutral toward the mineralization of carbofuran or linuron mediated by KN65.2 or SRS16, respectively, showing no difference in any kinetic parameter (Fig. 1). Despite the different effects of K169 on pesticide mineralization, as previously observed for MSH1, none of the pesticide catabolic strains were affected in cell density by K169 (Fig. 1d). On the other hand, the cell density of K169 (*D_t7,K169_*) was significantly higher (factor 2.1–2.8) in most of the dual-species systems compared to the K169 mono-species system (Fig. 1e and f). The exception was the dual-species system containing JMP134, where *D_t7,K169_* did not differ from this in the K169 mono-species system (Fig. 1e).

### Pesticide mineralization and cell density in mono- and dual-species systems at t_14_

**Fig 2:**
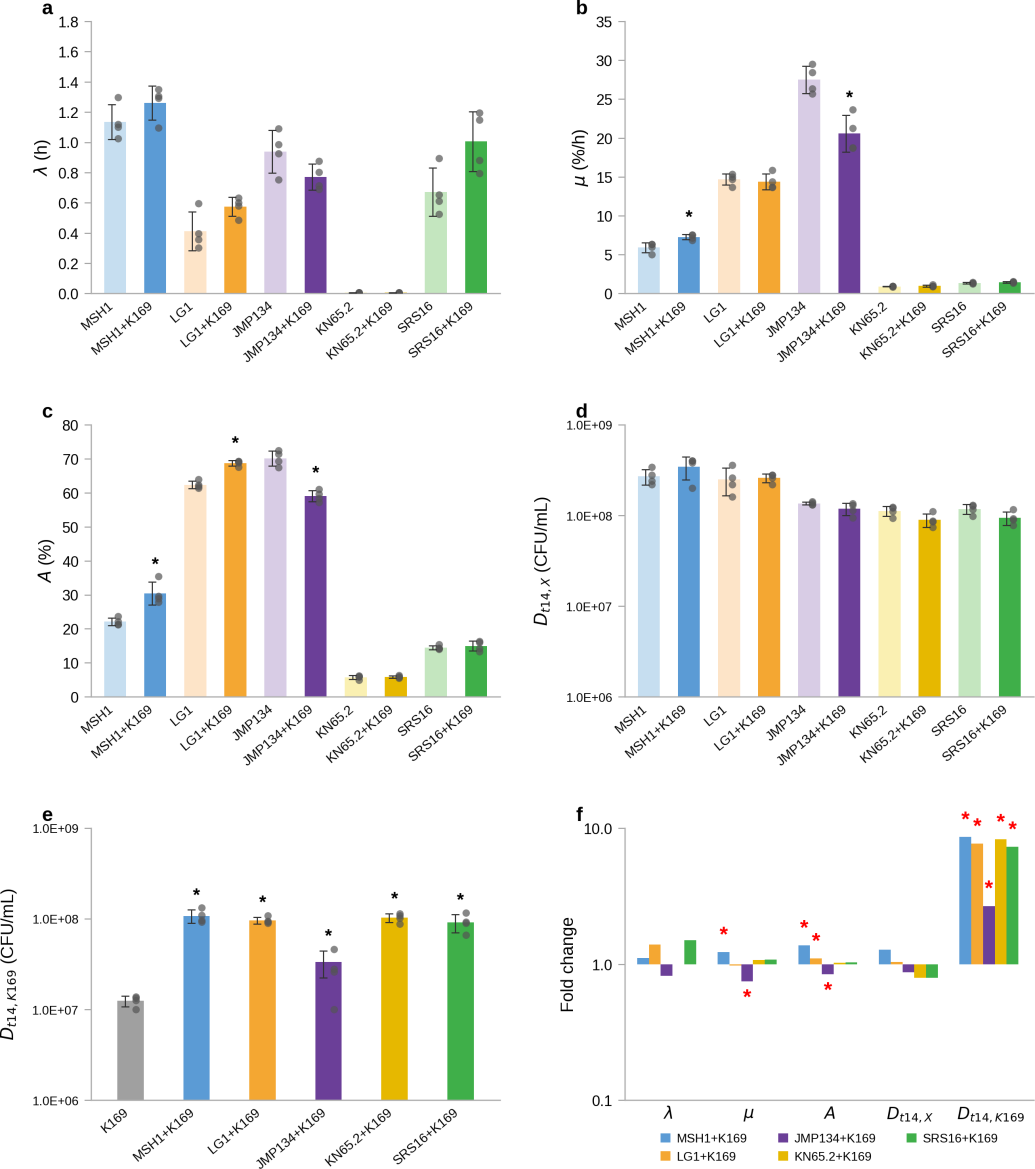
Values of pesticide mineralization parameters lag time (*λ*, panel **a**), maximum rate (*µ*, panel **b**), and extent (*A*, panel **c**) and cell densities of the tested pesticide catabolic bacteria (*D_t14,X_*, panel **d**) and K169 (*D_t14,K169_*, panel **e**) at t_14_ as observed in dual-species assemblies (*R_T_* = 2) of a pesticide catabolic strain and *Piscinibacter* sp. K169 and respective mono-species systems (*R_T_* = 1). Panel** f** shows the fold change of the respective parameters in the dual-species systems (*R_T_* = 2) compared to the respective mono-species systems (*R_T_* = 1). The values shown are averages with standard deviation (shown in error bar) based on four replicates, with the dots showing the values of each replicate. The black asterisk in panels a to e indicates significant differences between *R_T_* = 2 and *R_T_* = 1 systems containing the same degrader strain and/or K169 (*P*-value < 0.05). The red asterisk in panel f indicates values for which the difference between *R_T_* = 2 and *R_T_* = 1 systems was significant (*P*-value < 0.05). The color of each bar corresponds to those used for indicating the mineralization curves in Fig. S2 from which the respective parameter values were deduced.

In order to acquire knowledge on the longevity of the interaction displayed between K169 and pesticide degraders, mono- and dual-species microcosms were also incubated for 14 days (t_14_) and analyzed at t_14_, as done at t_7_. Pesticide mineralization curves are shown in Fig. S2. Values of *λ*, *µ*, *A*, and *D_t14,X_* are shown in Table S2 and graphically in Fig. 2a through e. Calculated fold changes in the respective parameters between dual-species and respective monoculture systems are shown in Table S3 and Fig. 2f. BAM mineralization mediated by MSH1 remained facilitated by the presence of K169, although the typical decrease in *λ* observed at t_7_ disappeared, while *μ* remained higher compared to the MSH1 mono-species system (Fig. 2a and b). However, *λ* increased compared to its value at t_7_ both in the MSH1 mono-species system (factor 2.4) and in the dual-species system (factor 4.9). *μ* did not change between t_7_ and t_14_, while *A* decreased in the mono-species system and increased in the dual-species system (both 1.3-fold) at t_14_ compared to t_7_. In contrast to t_7_, at t_14_, K169 exhibited a neutral effect on BAM mineralization by strain LG1, as neither *λ* nor *μ* differed between the dual-species system and the LG1 monoculture system (Fig. 2a and b). Between t_7_ and t_14_, *λ*, *μ,* and *A* did not change for the mono-species system and the dual-species system containing LG1, except for *λ* and *μ* at t_14_ in the dual-species system that increased (factor 1.5) and decreased (factor 1.4), respectively. The effect of K169 on 2,4-D mineralization by JMP134 became negative at t_14_ as *μ* in the dual-species system was lower than in the monoculture system (Fig. 2b), despite a remarkable increase of *μ* (factor 1.9) in the mono-species system at t_14_ compared to t_7_. *λ* (factor 1.5 in both the mono- and dual-species system) and *A* (factor 1.7, only in the JMP134 mono-species system) increased compared to the values at t_7_. As observed at t_7_, pesticide mineralization by KN65.2 and SRS16 remained unaffected by K169, as *λ*, *μ,* or *A* did not differ between the respective dual-species and mono-species systems (Fig. 2a, b and c). Nevertheless, compared to t_7_, kinetic parameters of carbofuran mineralization by KN65.2 did not change, while *λ* and *μ* of linuron mineralization by SRS16 decreased (factor 1.4–1.6) in both mono- and dual-species systems at t_14_. As observed at t_7_, despite the observed effects on pesticide mineralization for several strains, *D_t14,X_* values in dual-species assemblies for all strains were identical to those in the respective mono-species systems, suggesting a neutral effect of K169 on growth of the pesticide catabolic strains at t_14_ (Fig. 2d). *D_t14,MSH1_* and *D_t14,LG1_* did not change in neither of the combinations compared to the respective t_7_ values. In contrast, *D_t14,JMP134_, D_t14,KN65.2_*, and *D_t14,SRS16_* were factor 2.2–3.3 lower compared to, respectively, *D_t7,JMP134_, D_t7,KN65.2_*, and *D_t7,SRS16_* in both the dual- and mono-species systems. On the other hand, as for *D_t7,K169_*, *D_t14,K169_* was positively affected (factors 2–8) in the presence of each of the pesticide catabolic strains (Fig. 2e and f). Moreover, *D_t14,K169_* in the mono-species system decreased (factor 2.7) compared to *D_t7,K169_*, while *D_t14,K169_* in all dual-species systems remained the same.

## DISCUSSION

### Variable cooperation of *Piscinibacter* sp. K169 with other pesticide degraders

After the initial competition phase of 7 days, cooperative interactions, either mutual (both actors benefit) or unidirectional (one actor benefits), were observed between K169 and the different pesticide degraders. Besides MSH1, a mutual positive interaction with K169 was only observed with *A. niigataensis* LG1 mineralizing BAM, that is, as observed before for the MSH1-K169 assembly, K169 showed a positive effect on LG1-mediated BAM mineralization, while LG1 stimulated growth of K169. The observation that only the strain LG1, which taxonomically is most related to MSH1, that is, LG1, showed mutual facilitation with K169, suggests that the stimulation in both directions is rather genus/species or catabolic pathway specific, although more degraders belonging to other and the same taxa should be tested. However, beyond the two BAM catabolic *A. niigataensis* strains, K169 enhanced 2,4-D mineralization by the phylogenetically distant strain *C. pinatubonensis* JMP134, albeit without an impact of JMP134 on the growth of K169. That K169 is also able to support pesticide mineralization in other bacteria and taxa besides *A. niigataensis* MSH1 shows that the supporting effect of K169 can extend across degraders with different taxonomic backgrounds. These observations might be reminiscent of the concept of accidental interaction in which one species unintentionally impacts another, without incurring any additional cost, rather than being a dedicated interactive partnership that is triggered and had evolved between two partners (23). The latter would have made the interaction more specific and rarer and hence less likely to be discovered (23). We previously showed that two of the 13 sand filter isolates tested supported BAM mineralization by MSH1 in dual-species assemblies, representing a relatively high proportion of the isolates examined (22). Furthermore, the observation that also 2,4-D catabolism in JMP134 was stimulated by K169 shows that the interaction and the underlying mechanism(s) of the effect of K169 toward MSH1/LG1 is not pathway specific, especially since both pathways quite differ in catalytic and enzymatic make-up (10, 36). While we cannot exclude that some of the enzymes involved share common requirements delivered by K169, and/or that K169 uses different mechanisms to stimulate the two pathways, a more plausible hypothesis is that K169 rather affects the overall cellular metabolism and physiology of the degraders than the pathway itself. This hypothesis is supported by the observation that as previously shown for the MSH1-K169 partnership, stimulation of LG1 and JMP134 mediated pesticide mineralization was not accompanied by increased pesticide catabolic strain cell densities (compared to the respective mono-species systems). Hence, the impact on overall cell physiology and stimulating cell activity rather than cell density appears to be the common way that K169 positively impacts pesticide mineralization. Previous studies have shown that in pure bacterial cultures, the kinetics of pollutant degradation not only depend on cell densities but also on the physiological state of the cells (37–39), while the incubation of MSH1 under oligotrophic conditions was shown to affect the specific BAM degradation and mineralization rate (16, 40). Moreover, this is consistent with a broader body of work showing that non-degrading or partner organisms can enhance degradation performance without necessarily increasing the abundance of the catalytic strains (16, 40). The mechanism underlying the supporting impact of K169 on cell physiology of and pesticide mineralization by MSH1, LG1, and JMP134 remains to be elucidated. Studies in other systems have attributed comparable helper effects to physiological support such as provision of limiting cofactors or nutrients (18, 20, 41, 42), removal of waste products (19), and/or mitigation of inhibitory compounds (43, 44). While the supporting impact of K169 toward pesticide mineralization was observed with three of the five tested catabolic strains (including MSH1), four of the strains supported growth of K169. Although the pesticide-catabolic strains might provide additional organic carbon by cross-feeding, a more plausible explanation is that K169 lacks an essential factor for proper growth and that that factor is synthesized and excreted by many types of bacteria. In support of this, we have observed before that K169, isolated and propagated on R2A, does not grow on the minimal medium used in this study when supplemented with any of the single carbon sources present in R2A or any other carbon source tested (45). Elucidating the underlying mechanisms of cooperation is a crucial next step in understanding the interactions of the pesticide degraders with K169 in both directions and warrant targeted follow-up work and dedicated methodologies such as conditioned-medium assays, membrane-separated co-cultures, metabolomics, and/or targeted metabolite or cofactor supplementation (19, 42, 46–48).

Another observation of interest was that neither K169 nor any of the pesticide catabolic strains did negatively affect its partner in dual-species systems either in terms of pesticide mineralization or growth. This suggests a limited capacity of exploitative and/or interference competition from the side of K169 toward the tested pesticide catabolic strains and vice versa. Foster and Bell (49) reported that within a microbial community, competition is more frequent than positive interactions, whereas Ren et al. (26) reported the opposite. In both studies, it concerned bacterial isolates obtained from and hence co-existing in the same environment, which is not the case in our study. Previously, 8 of the 13 tested sand filter isolates negatively impacted MSH1-mediated BAM mineralization (22). Based on our studies so far, K169 and the pesticide catabolic strains largely depend on other resources and fill in different niches in the sand microcosm system, and both exploitative and interference competition between the two partners is limited.

The observation that K169 stimulated OMP mineralization in other micropollutant degraders different from MSH1 is of interest for bioaugmentation purposes aiming at promoting pollutant biodegradation in DWTPs for drinking water cleansing, since it suggests that K169 can be used to support bioaugmentation of DWTP systems that face other OMPs than BAM by inoculating other degraders. However, in contrast to MSH1 and LG1, JMP134 did not stimulate growth of K169. Hence, the fitness of JMP134 might be less tied to that of K169 than in the case of the MSH1/K169 and LG1/K169 partnerships, and JMP134 will not profit from the growth of K169 and the possible additional benefits it gives, such as improved spatial segregation from other competitive organisms present in the resident community of DWTPs (50, 51). In addition, the indication that K169 supports co-bioaugmentation for mixed OMPs is relevant, since pollution of groundwater resources with multiple micro-pollutants is recurrent (52, 53). In that scenario, however, the question remains in which way the multiple degrader inocula will compete for the services profited by K169 (54). Broad specificity “helper” effects have been reported in pollutant-degrading consortia (17, 55), and degrader inocula that are unidirectionally stimulated by K169 (like JMP134) might profit from degrader inocula that show mutual positive effects with K169 and increase the K169 cell density. Moreover, the different inoculum strains might engage in secondary interactions such as rock-paper-scissors relationships or higher-order interactions that affect the pairwise interaction between each of the degraders and K169 (56–58). Evidence from other engineered biodegradation settings shows that outcomes of combining multiple inocula can be strongly shaped by community-level interactions (59, 60). Finally, the observation that K169 growth was never negatively impacted by any of the catabolic strains and was even stimulated by the majority of the tested strains might suggest that other bacteria in any DWTP target community might stimulate K169 growth. We previously showed that the impact of K169 on MSH1 functionality remained when the MSH1-K169 partnership was applied in artificial higher richness communities (61). The K169 cell density was, though, not determined.

### Beneficial effect of K169 on pesticide-degrading strains deteriorates with time

After 2 weeks of interaction, at t_14_, the beneficial effect of K169 on pesticide mineralization became weak in the case of MSH1 and even completely disappeared in the case of LG1. Moreover, 2,4-D mineralization mediated by JMP134 became negatively affected by the presence of K169. However, cell densities of the degrader strains in dual-species systems remained similar to those in the mono-species systems and did not change between t_7_ and t_14_. Similarly, cell densities of K169 remained higher in the dual-species systems compared to the mono-species systems and did not deteriorate between t_7_ and t_14_. The discrepancies between pesticide mineralization parameters and cell densities at t_14_ compared to t_7_ indicate that the deterioration of the K169 beneficial effects is due to the loss of cell activities rather than cell numbers. Possibly, at t_14_, the organisms experienced more and/or longer starvation conditions than at t_7_ due to changes in nutrient conditions, and the activity of K169 may deteriorate in such a way that factors delivered by K169 to stimulate pesticide mineralization in MSH1, LG1, and JMP134 could not be produced anymore or that inhibiting waste products were not removed anymore. Differences in nutrient context have been shown before to impact interaction outcomes between microorganisms while resource limitation shifted the nature of interactions within the same partner pair from facilitative to neutral or even negative (62–64). Previous studies that examine the bacterial response to nutrient starvation show that many bacteria experience a severe reduction (up to 90%) in cellular RNA content (36), suggesting a reduced production or consumption of metabolites. Otherwise, the prolonged starvation conditions may have affected the pesticide degrader cells in such a way that they were not accessible anymore to the stimulating factors provided by K169. It was previously shown in laboratory and pilot conditions that BAM mineralization by MSH1 deteriorates with time despite maintaining cell densities (13, 14, 16). The fact that none of the partners lost substantial cell densities suggests that the cells were still viable and culturable on the detection media despite the starvation conditions. A reason for the starvation at t_14_ is likely the exhaustion of the organic carbon on the sand, which serves as the main carbon source driving the interactions between MSH1 and K169, and likely also in the other K169-pesticide catabolic strain partnerships (27). At t_14_, the effect of K169 on JMP134 even became negative. This poses questions about the finiteness of the organic carbon present in the actual target sand filters exploited in DWTPs in the case of applying bioaugmentation. Sand filters exploited in DWTPs are continuous and dynamic systems; that is, there is a continuous supply of new carbon with the incoming water, although DOC concentrations in groundwater and hence input water for sand filters in DWTPs are relatively low (65–68). Otherwise, heterotrophs like K169 and catabolic OMP degraders might be sustained by DOC originating from the activity or decay of chemolithotrophs that utilize inorganic electron donors that are plentiful in the intake waters (69). However, the varying types of sand filters, reactors, and water conditions can result in different nutrient conditions, thereby influencing the impact of K169 on pesticide-degrading organisms and the longevity of their interactions. Clearly, there is a need to understand the sources, metabolic and anabolic networks, and turnover of organic carbon in sand filters in DWTPs to fully manage any OMP degrader-K169 partnership for optimized bioaugmentation. Overall, our findings show that microbial species that are not directly involved in pesticide catabolism may nevertheless play a critical role in regulating degradation processes through interaction-mediated effects on degrader activity.

## SUPPLEMENTAL MATERIAL

Supplemental material aem.00093-26-s0001.docx Fig. S1 and S2; Tables S1 to S3.

ASM does not own the copyrights to Supplemental Material that may be linked to, or accessed through, an article. The authors have granted ASM a non-exclusive, world-wide license to publish the Supplemental Material files. Please contact the corresponding author directly for reuse.
