# Supplementary material for: Specificity and longevity of a bacterial interspecies mutual cooperation benefiting organic micropollutant biodegradation
Source: Appl Environ Microbiol. 2026 Jul 1;92(7):e00093-26. doi: 10.1128/aem.00093-26 (PMC13390472; doi:10.1128/aem.00093-26)
Supplement: Supplemental material — Fig. S1 and S2; Tables S1 to S3. [file aem.00093-26-s0001.docx]

Table S1. Growth media and growth periods applied for pre-culturing the bacterial strains used in this study.

| Strain | Pesticide | Agar medium | Incubation period (days) | Liquid medium | Incubation period (days) |
| --- | --- | --- | --- | --- | --- |
| *Piscinibacter sp. K169* | \ | R2A^1^ | 7 | R2B^2^ | 3 |
| *A. niigataensis* MSH1 | BAM | R2A + BAM (100 mg/L) + Km (50 mg/L) | 7 | R2B + BAM (10 mg/L) | 2 |
| *A. niigataensis* LG1 | BAM | R2A + BAM (100 mg/L) + Km (50 mg/L) | 7 | R2B + BAM (10 mg/L) | 2 |
| *A. necator* JMP134 | 2,4-D | TSA^3^ | 2 | TSB^4^ | 1 |
| *Novosphingobium* sp. KN65.2 | Carbofuran | TSA^3^ | 2 | TSB^4^ | 1 |
| *Variovorax* sp. SRS16 | Linuron | R2A^1^ | 5 | R2B^2^ | 2 |

References：

1. For preparation of R2A (R2B containing agar - used as solid agar medium), 14 g Select Agar (Invitrogen, Carlsbad, CA, USA) was added per liter medium.
2. Reasoner, D. J., and Geldreich, E. E. (1985) A new medium for the enumeration and subculture of bacteria from potable water, *Appl Environ Microbiol* *49*, 1–7.
3. For preparation of TSA (TSB containing agar - used as solid agar medium), 14 g Select Agar (Invitrogen, Carlsbad, CA, USA) was added per liter medium.
4. Tryptone soya broth (TSB, Oxoid)

| 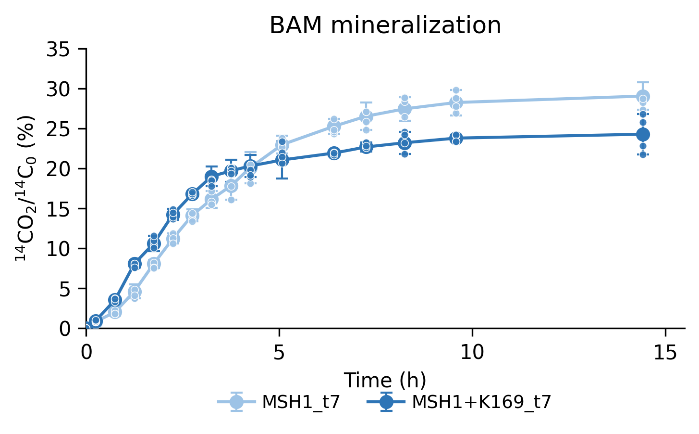  a | 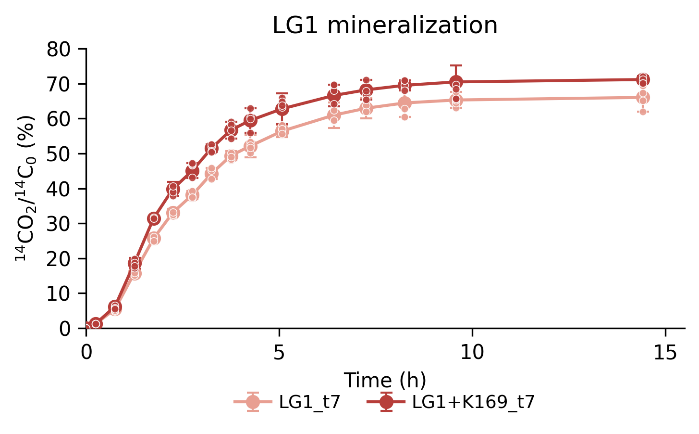  b |
| --- | --- |
| 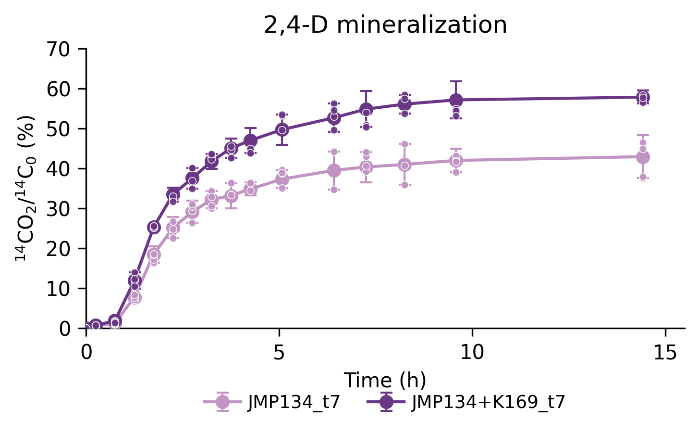  c | 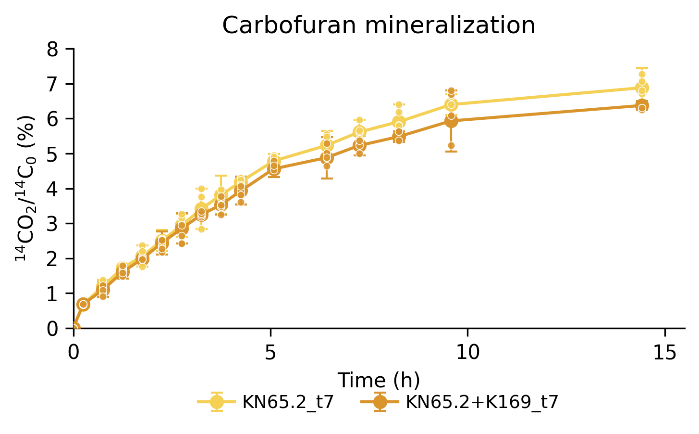  d |
| 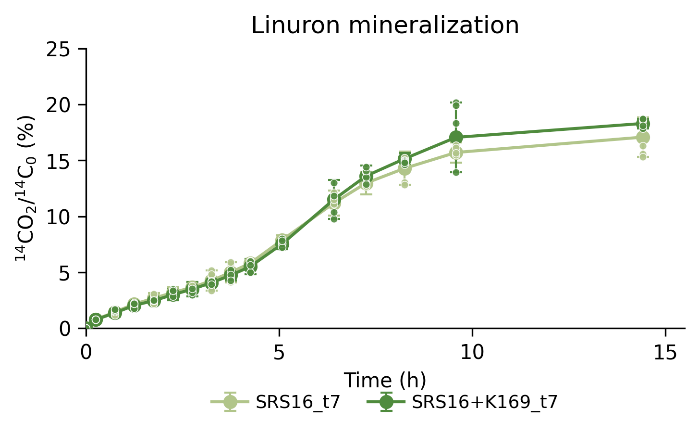  e | Figure S1. Cumulative pesticide mineralization curves at t_7_ (cumulative ^14^CO_2_ production relative to the initial amount of ^14^C-pesticide added (^14^C_0_)) obtained for dual-species assemblies of a pesticide catabolic strain and *Piscinibacter* sp. K169 (*R_T_* = 2) and for the respective pesticide catabolic strain in a mono-species system (*R_T_* = 1). The different colours refer to the mono-species and dual-species systems as indicated below the graph. The values shown are averages with standard deviation (shown in error bar) based on four replicates with the dots showing the values of each replicate. The time points at which ^14^CO_2_ production/accumulation was determined were 0.25, 0.75, 1.25, 1.75, 2.25, 2.75, 3.25, 3.75, 4.25, 5.08, 6.42, 7.25, 8.25, 9.58, 14.42, 28, 53.58, 104 and 130 (h) but the graphs only show cumulative mineralization for 15 hours since all curves reached maximum cumulative mineralization after 15 hours of incubation. The color of each curve corresponds to the bar under the same condition in Figure 1. |

| 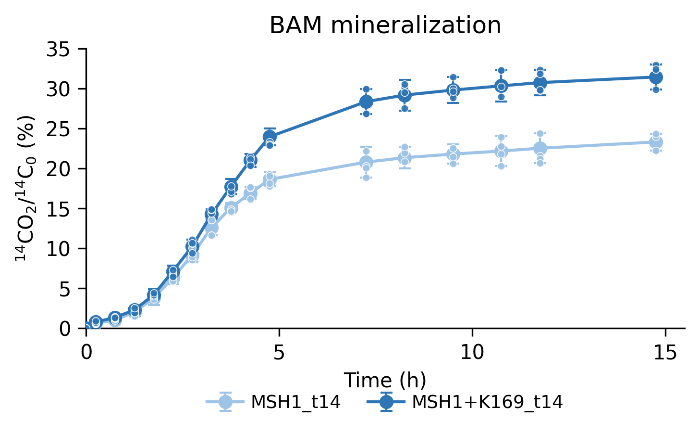  a | 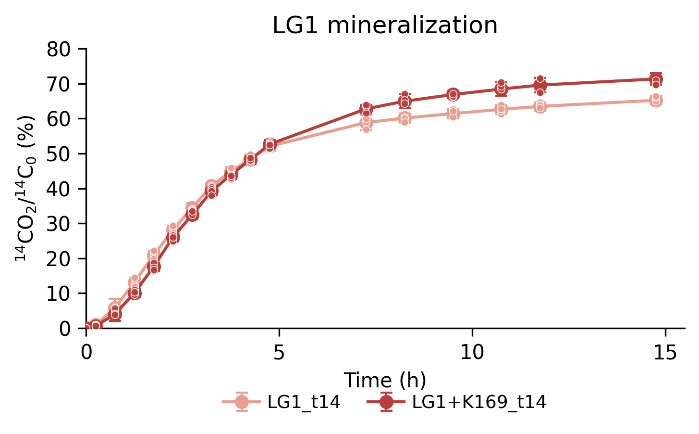  b |
| --- | --- |
| 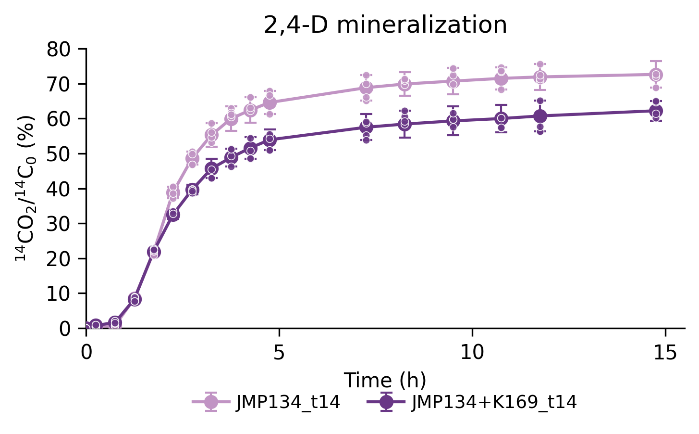  c | 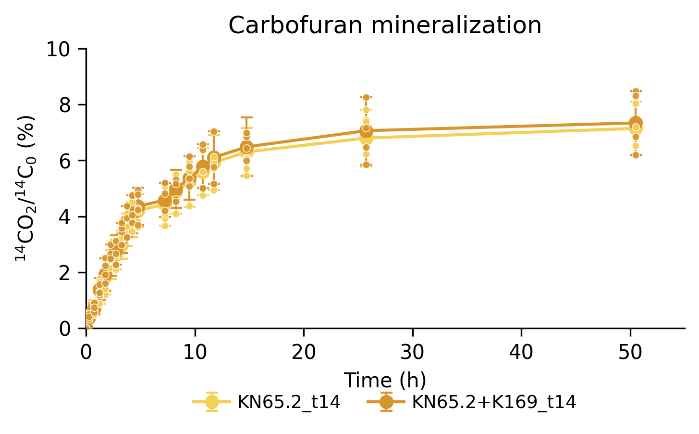  d |
| 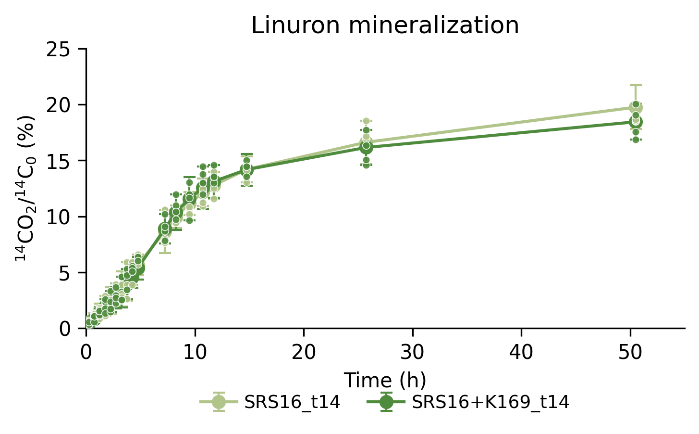  e | Figure S2. Cumulative pesticide mineralization curves at t_14_ (cumulative ^14^CO_2_ production relative to the initial amount of ^14^C-pesticide added (^14^C_0_)) obtained for dual-species assemblies consisting of a pesticide catabolic strain and *Piscinibacter* sp. K169 (*R_T_* = 2) and for the respective pesticide catabolic strain in mono-species systems (*R_T_* = 1). The different colours refer to the mono-species and dual-species systems as indicated below the graph. The values shown are averages with standard deviation (shown in error bar) based on four replicates with the dots showing the values of each replicate. The time points at which ^14^CO_2_ production/accumulation was determined at t_14_ were 0.25, 0.75, 1.25, 1.75, 2.25, 2.75, 3.25, 3.75, 4.25, 4.75, 7.25, 8.25, 9.5, 10.75, 11.75, 14.75, 25.75, 50.5, 99.25 and 130 (h), but the graphs only show cumulative mineralization for 50.5 hours since all curves reached maximum cumulative mineralization after 50.5 hours of incubation. The color of each curve corresponds to the bar under the same condition in Figure 2. |

Table S2. Average values ± standard deviations (from four replicates) of pesticide mineralization parameters *λ*, *µ* and *A*, and of the cell densities Dt7,X, Dt7,K169, Dt14,X and Dt14,K169 obtained for dual-species systems consisting of *Piscinibacter* sp. K169 and one of the tested pesticide catabolic strains (*R_T_* = 2), and for mono-species systems of K169 or the respective pesticide catabolic strains (*R_T_* = 1). The green number indicates a significant positive effect of K169 on the pesticide mineralization while the red number indicates a significant negative effect of K169 on the pesticide mineralization parameter or cell densities. The black asterisk indicates significant different parameter values between dual-species (*R_T_* = 2) and mono-species systems (*R_T_* = 1) for K169 or a specific pesticide degrader.

|  | ***λ* (h)** | ***µ* (%/h)** | ***A* (%)** | ***D_X_* (CFU/mL)** | ***D_K169_* (CFU/mL)** |
| --- | --- | --- | --- | --- | --- |
| **t_7_** |  |  |  |  |  |
| **MSH1** | 0.46 ± 0.051 | 5.83 ± 0.15 | 28.17 ± 1.43 | 3.3^E+8^ ± 7.4^E+7^ | \ |
| **K169** | \ | \ | \ | \ | 3.4^E+7^ ± 5.2^E+6^ |
| **MSH1+K169** | 0.26 ± 0.015* | 7.21 ± 0.17* | 22.79 ± 1.00* | 3.4^E+8^ ± 7.1^E+7^ | 7.2^E+7^ ± 1.5^E+7^* |
| **LG1** | 0.36 ± 0.016 | 16.41 ± 0.90 | 64.31 ± 1.64 | 3.0^E+8^ ± 4.3^E+7^ | \ |
| **LG1+K169** | 0.37 ± 0.024 | 20.30 ± 0.79* | 68.87 ± 2.82 | 3.4^E+8^ ± 1.7^E+8^ | 9.7^E+7^ ± 1.9^E+7^* |
| **JMP134** | 0.61 ± 0.023 | 14.20 ± 1.05 | 40.33 ± 2.10 | 4.5^E+8^ ± 1.0^E+8^ | \ |
| **JMP134+K169** | 0.52 ± 0.030 | 17.63 ± 1.01* | 54.90 ± 2.28* | 3.6^E+8^ ± 9.4^E+7^ | 3.0^E+7^ ± 1.3^E+7^ |
| **KN65.2** | 0.01 ± 0.001 | 1.03 ± 0.05 | 6.50 ± 0.21 | 3.0^E+8^ ± 7.2^E+7^ | \ |
| **KN65.2+K169** | 0.01 ± 0.001 | 0.98 ± 0.02 | 5.97 ± 0.30 | 2.0^E+8^ ± 9.5^E+7^ | 7.8^E+7^ ± 7.5^E+6^* |
| **SRS16** | 1.07 ± 0.164 | 2.10 ± 0.11 | 16.49 ± 1.26 | 3.2^E+8^ ± 7.5^E+7^ | \ |
| **SRS16+K169** | 1.40 ± 0.144 | 2.28 ± 0.14 | 19.55 ± 1.49 | 2.1^E+8^ ± 1.0^E+8^ | 7.5^E+7^ ± 2.4^E+7^* |
| **t_14_** |  |  |  |  |  |
| **MSH1** | 1.14 ± 0.115 | 5.91 ± 0.63 | 22.10 ± 1.11 | 2.7^E+8^ ± 5.3^E+7^ | \ |
| **K169** | \ | \ | \ | \ | 1.2^E+7^ ± 1.7^E+6^ |
| **MSH1+K169** | 1.26 ± 0.113 | 7.26 ± 0.32* | 30.46 ± 3.39* | 3.5^E+8^ ± 9.7^E+7^ | 1.1^E+8^ ± 1.8^E+7^* |
| **LG1** | 0.41 ± 0.128 | 14.67 ± 0.71 | 62.36 ± 1.10 | 2.5^E+8^ ± 8.4^E+7^ | \ |
| **LG1+K169** | 0.57 ± 0.063 | 14.38 ± 1.04 | 68.70 ± 0.81* | 2.6^E+8^ ± 2.8^E+7^ | 9.6^E+7^ ± 8.2^E+6^* |
| **JMP134** | 0.94 ± 0.142 | 27.49 ± 1.77 | 70.14 ± 2.18 | 1.4^E+8^ ± 4.9^E+6^ | \ |
| **JMP134+K169** | 0.77 ± 0.086 | 20.58 ± 2.37* | 59.06 ± 1.65* | 1.2^E+8^ ± 1.9^E+7^ | 3.3^E+7^ ± 1.1^E+7^* |
| **KN65.2** | 0.01 ± 0.001 | 0.91 ± 0.04 | 5.75 ± 0.54 | 1.1^E+8^ ± 1.3^E+7^ | \ |
| **KN65.2+K169** | 0.01 ± 0.001 | 0.97 ± 0.12 | 5.88 ± 0.35 | 8.9^E+7^ ± 1.5^E+7^ | 1.0^E+8^ ± 1.1^E+7^* |
| **SRS16** | 0.67 ± 0.159 | 1.34 ± 0.08 | 14.48 ± 0.62 | 1.2^E+8^ ± 1.5^E+7^ | \ |
| **SRS16+K169** | 1.01 ± 0.197 | 1.45 ± 0.09 | 14.95 ± 1.44 | 9.4^E+7^ ± 1.6^E+7^ | 9.1^E+7^ ± 2.0^E+7^* |

Table S3. Calculated fold changes of the pesticide mineralization parameters *λ*, *µ* and *A*, and of the cell densities Dt7,X, Dt7,K169, Dt14,X and Dt14,K169 between dual-species and mono-species systems in sand microcosms with *Piscinibacter* sp. K169 and one of five pesticide catabolic strains. The black asterisk indicates significant different parameter values between dual-species (*R_T_* = 2) and mono-species systems (*R_T_* = 1) for K169 or a specific pesticide degrader.

|  | ***λ* (h)** | ***µ* (%/h)** | ***A* (%)** | ***D_X_* (CFU/mL)** | ***D_K169_* (CFU/mL)** |
| --- | --- | --- | --- | --- | --- |
| **t_7_** |  |  |  |  |  |
| **MSH1+K169** | 0.55* | 1.24* | 0.81* | 1.03 | 2.10* |
| **LG1+K169** | 1.05 | 1.24* | 1.07 | 1.12 | 2.84* |
| **JMP134+K169** | 0.85 | 1.24* | 1.36* | 0.81 | 0.88 |
| **KN65.2+K169** | 1.00 | 0.95 | 0.92 | 0.67 | 2.29* |
| **SRS16+K169** | 1.31 | 1.09 | 1.19 | 0.67 | 2.19* |
| **t_14_** |  |  |  |  |  |
| **MSH1+K169** | 1.11 | 1.23* | 1.38* | 1.28 | 8.63* |
| **LG1+K169** | 1.39 | 0.98 | 1.10* | 1.04 | 7.71* |
| **JMP134+K169** | 0.82 | 0.75* | 0.84* | 0.87 | 2.68* |
| **KN65.2+K169** | 1.00 | 1.07 | 1.02 | 0.80 | 8.27* |
| **SRS16+K169** | 1.50 | 1.08 | 1.03 | 0.80 | 7.31* |
